# Interaction between genetic risk score and dietary fat intake on lipid-related traits in Brazilian young adults

**DOI:** 10.1017/S0007114524001594

**Published:** 2024-09-14

**Authors:** Ramatu Wuni, Heyam Amerah, Serena Ammache, Nathália T. Cruvinel, Nara R. da Silva, Gunter G. C. Kuhnle, Maria A. Horst, Karani S. Vimaleswaran

**Affiliations:** 1 Hugh Sinclair Unit of Human Nutrition, Department of Food and Nutritional Sciences and Institute for Cardiovascular and Metabolic Research (ICMR), University of Reading, Reading RG6 6DZ, UK; 2 Nutritional Genomics Research Group, Faculty of Nutrition, Federal University of Goiás (UFG), Goiania, Brazil; 3 Institute for Food, Nutrition, and Health (IFNH), University of Reading, Reading RG6 6EU, UK

**Keywords:** Genetic risk score, Brazil, TAG to HDL-cholesterol ratio, Fat intake, SFA

## Abstract

The occurrence of dyslipidaemia, which is an established risk factor for cardiovascular diseases, has been attributed to multiple factors including genetic and environmental factors. We used a genetic risk score (GRS) to assess the interactions between genetic variants and dietary factors on lipid-related traits in a cross-sectional study of 190 Brazilians (mean age: 21 ± 2 years). Dietary intake was assessed by a trained nutritionist using three 24-h dietary recalls. The high GRS was significantly associated with increased concentration of TAG (beta = 0·10 mg/dl, 95 % CI 0·05–0·16; *P* < 0·001), LDL-cholesterol (beta = 0·07 mg/dl, 95 % CI 0·04, 0·11; *P* < 0·0001), total cholesterol (beta = 0·05 mg/dl, 95 % CI: 0·03, 0·07; *P* < 0·0001) and the ratio of TAG to HDL-cholesterol (beta = 0·09 mg/dl, 95 % CI: 0·03, 0·15; *P* = 0·002). Significant interactions were found between the high GRS and total fat intake on TAG:HDL-cholesterol ratio (*P*_interaction_ = 0·03) and between the high GRS and SFA intake on TAG:HDL-cholesterol ratio (*P*_interaction_ = 0·03). A high intake of total fat (>31·5 % of energy) and SFA (>8·6 % of energy) was associated with higher TAG:HDL-cholesterol ratio in individuals with the high GRS (beta = 0·14, 95 % CI: 0·06, 0·23; *P* < 0·001 for total fat intake; beta = 0·13, 95 % CI: 0·05, 0·22; *P* = 0·003 for SFA intake). Our study provides evidence that the genetic risk of high TAG:HDL-cholesterol ratio might be modulated by dietary fat intake in Brazilians, and these individuals might benefit from limiting their intake of total fat and SFA.

CVD are a top cause of mortality globally, accounting for 32 % of all deaths worldwide in 2019^(1)^. Over three-quarters of mortality from CVD has been reported to occur in low- and middle-income countries^(1)^, highlighting the enormous impact of CVD in these countries. In Brazil, ischaemic heart disease and stroke accounted for most deaths in 2019, with a percentage increase of 18 and 14 %, respectively, from 2009^(2)^. An analysis of the factors contributing to death in Brazil using data from the Global Burden of Disease 2019 study^(3)^ indicated that, more than 80 % of deaths from CVD is attributable to cardiovascular risk factors. Among the risk factors for CVD is an altered blood lipid profile (dyslipidaemia), which is evidenced by a rise in the concentration of triacylglycerol (TAG) or LDL-cholesterol and a reduction in the concentration of HDL-cholesterol^(4,5)^.

The occurrence of dyslipidaemia has been attributed to multiple factors including genetic and environmental factors^(6–11)^. Dietary fatty acids are involved in modulating the metabolism of lipids and lipoproteins^(12,13)^, and dietary recommendations to reduce CVD risk advocate for a reduction in SFA and total fat intake^(14)^. A high SFA intake has been associated with a rise in TAG-rich lipoproteins, which is associated with increased risk of myocardial infarction, ischaemic stroke, and other CVD^(15–17)^. Consumption of SFA has also been linked to a rise in circulating levels of inflammatory biomarkers^(18,19)^ which contributes to the development of cardiometabolic diseases, including CVD^(20–22)^. A meta-analysis involving a total of forty-nine prospective studies^(23)^ identified that higher concentration of circulating SFA was associated with a 50 % increased risk of CVD, 63 % increased risk of CHD and 38 % increased risk of stroke. In a cross-sectional study of 282 Brazilian adults^(24)^, consumption of SFA was found to be higher than the recommended intake in 79·7 % of the participants. The fat content of processed foods in Brazil was also found to be composed of high amounts of SFA, ranging from 9·3 to 12 g per 100 g of food products^(25)^.

Evidence from genome-wide association studies has implicated several genetic loci for the development of dyslipidaemia^(26–30)^, but these variants account for a small proportion of variability in blood lipid concentrations, and there is growing evidence that an interaction between genetic variants and environmental factors is responsible for part of the missing heritability^(31–36)^. Single variants often have small effect sizes and an effective approach to assessing the genetic contribution to complex traits is the use of a genetic risk score (GRS), which allows the combined effect of multiple variants to be analysed^(37,38)^. Single nucleotide polymorphisms (SNP) of lipid-pathway genes have been reported to contribute to variations in blood lipid concentrations^(7,39–41)^, and the proteins encoded by these genes include cholesteryl ester transfer protein (CETP), which regulates HDL-cholesterol concentration and particle size by promoting the transfer of cholesteryl esters and TAG between lipoproteins^(42)^; apolipoprotein A1 (APOA1), which is the main component of HDL-cholesterol and is involved in the maturation of HDL-cholesterol^(43)^; glucokinase regulatory protein, which regulates the activity of glucokinase^(44,45)^; sortilin, which regulates plasma LDL-cholesterol by facilitating hepatic uptake of ApoB100-containing lipoproteins^(46)^ and hepatic lipase (LIPC) and endothelial lipase (LIPG) which hydrolyse lipoproteins to release free fatty acids^(47,48)^. Only a few studies have utilised a GRS to assess the interactions between dietary intake and genetic variants on CVD traits in Brazilians^(37,49,50)^, with even fewer studies focusing on young adults. Two of the studies^(37,49)^ used data from the Obesity, Lifestyle and Diabetes in Brazil (BOLD) cross-sectional study and involved 187 and 200 participants aged 19–24 years, respectively. Significant GRS–diet interactions were found in relation to vitamin D and glycaemic traits, respectively. The third study^(50)^, which was also a cross-sectional study, consisted of 228 adults (19–60 years) and significant GRS–diet interactions on dyslipidaemia were reported. Hence, the aim of this study was to assess the genetic associations and the interaction of the GRS with dietary factors on lipid-related traits in Brazilian young adults.

## Methods

### Study participants

The study consisted of 190 young adults aged 19–24 years from the BOLD cross-sectional study^(34,37)^. Participants were recruited between March and June 2019 from the Federal University of Goiás. The study was performed as part of the gene–nutrient interactions (GeNuIne) collaboration, which is aimed at investigating how genetic and lifestyle factors interact to influence chronic diseases in diverse ethnic groups, with the goal of preventing and managing chronic diseases through personalised nutrition^(6,51–53)^. Details of the study design are published elsewhere^(37,49)^. In brief, a total of 416 individuals expressed interest in the study, but 207 individuals were found to be eligible. Participants were excluded if they were using lipid-lowering medication, vitamins or mineral supplements; had undergone dietary interventions in the past 6 months or undertaking vigorous physical activity or had a diagnosis of any chronic disease such as type 2 diabetes, dyslipidaemia or hypertension. Out of the 207 eligible participants, 200 completed the study; however, 190 participants were included in the present analysis after excluding participants with missing data for genetic and phenotypic measurements. The selection of the participants is shown in online Supplementary Fig. S1.

The study was approved by the Ethics Committee of the Federal University of Goiás (protocol number 3·007·456, 08/11/2018), and written informed consent was obtained from all the study participants. The study was performed in accordance with the ethical principles in the Declaration of Helsinki.

### Anthropometric and biochemical measurements

Measurement of anthropometric parameters was done by trained staff from the Nutritional Genomics research group of The Federal University of Goiás, Brazil. A Tanita® (Tanita Corporation) portable electronic scale, which has a maximum capacity of 150 kg, was used to weigh participants. For height, a stadiometer with a movable rod was used, and the volunteers were asked to keep upright with heels, calves, shoulder blades and shoulders pressed against the wall, knees straight, feet together and arms extended along the body; the head raised (making a 90º angle with the ground), with the eyes looking at a horizontal plane ahead, in accordance with the Frankfurt plane. Weight and height were used to calculate the BMI using the formula: weight (kg)/the square of the height (m^2^). Waist circumference was measured using an inelastic measuring tape at the midpoint between the lowest rib margin and the iliac crest^(54)^.

Blood pressure was measured when the patient was seated, positioning the arm at heart level. Three measurements were taken, with 5-min intervals between them. At the end, the average of the three measurements was considered, as proposed by the American Heart Association^(55)^ and approved by the VI Brazilian Guideline on Hypertension^(54)^.

Approximately 10 ml of venous blood was collected from the medial cubital vein following a 12-h fasting period. The blood collection procedure was performed by a trained healthcare professional using single-use materials. Participants were instructed to abstain from consuming alcohol for 72 h and avoid engaging in strenuous physical activity for 24 h prior to the blood collection. The samples were processed immediately after collection at the Romulo Rocha Laboratory (Goiânia, Brazil). The levels of TAG, total cholesterol (TC) and HDL-cholesterol were assessed using direct enzymatic colorimetry. LDL-cholesterol levels were calculated using the Friedewald, Levy, and Fredrickson equation (1972)^(56)^.

### Dietary assessment

Dietary intake was assessed by a trained nutritionist using three 24-h dietary recalls consisting of non-consecutive days, including one weekend^(57)^. The nutritionist conducted the first interview in person according to multiple-pass method^(58)^, and the following two interviews were conducted via phone calls. To assist in estimating portion sizes of various foods, participants were provided with measuring equipment such as measuring cups and spoons. Intake of nutrients and energy was determined from the dietary recalls using the Avanutri Online® diet calculation software (Avanutri Informática Ltda) with three Brazilian food composition databases, Brazilian Institute of Geography and Statistics, 2011^(59)^, food composition table-support for nutritional decision making (2016)^(60)^ and food studies and research centre-Brazilian food composition table (2011)^(61)^. For processed or ultra-processed foods that were not in the databases, the information in the label was manually added.

### Single nucleotide polymorphism selection and genotyping

A total of seven SNP representing seven loci were selected for this study based on their association with lipid-related traits at a genome-wide significance level (*P* < 5 × 10^–8^): *CETP* SNP rs3764261^(26,62–66)^, glucokinase regulator (*GCKR*) SNP rs1260326^(26,41,65,67–70)^, endothelial lipase (*LIPG*) SNP rs7241918^(26,71–73)^, sortilin 1 (*SORT1*) SNP rs629301^(26,71,72)^, hepatic lipase (*LIPC*) SNP rs1532085^(26,65,70,74)^, apolipoprotein A1 (*APOA1*) SNP rs964184^(26,27,68,75–79)^ and ATPase plasma membrane Ca2+ transporting 1 (*ATP2B1*) SNP rs2681472^(80–83)^. Table 1 shows the SNP, effect sizes, *P*-values and the genome-wide association studies. A review by our team^(7)^ indicated that the *CETP* gene had the highest number of reported associations with lipid traits, and it was concluded that SNP of the *CETP* gene could potentially alter blood lipid profiles by interacting with diet. The *GCKR* gene was chosen as it has been reported to influence alterations in blood lipid profiles^(90–95)^. The *LIPG* gene, another key lipid metabolism gene has been reported to play a role in inflammation and could influence the risk of CVD^(48,96,97)^. Furthermore, the *SORT1* gene is considered the strongest genome-wide LDL-cholesterol associated locus^(27,62,98–101)^ and the *LIPC* gene is also a main lipid-pathway gene which has been associated with abnormal lipid profiles^(26,65,72,74,88)^. Additionally, the *APOA1* gene has been widely studied and has been linked with variations in blood lipid levels^(26,28,76,78,85)^ and the risk of CVD^(102–105)^. Similarly, the *ATP2B1* gene has been reported to influence the risk of developing CVD^(80,81,83,89,104)^. Six of the SNP included in our GRS (rs3764261, rs1260326, rs7241918, rs629301, rs1532085, rs964184) had previously been included in a GRS by a genetic association study involving 6358 participants from the Multi-Ethnic Study of Atherosclerosis Classic cohort^(106)^ which observed significant associations between the GRS and lipid traits. The genotyping procedure has been previously published^(49)^. Briefly, blood samples (3 ml each) for genotyping were collected in BD Vacutainer® ethylenediamine tetraacetic acid (EDTA) tubes and kept at a controlled temperature of –80ºC during transportation by the World Courier Company. Genotyping was performed by LGC Genomics, London, UK (http://www.lgcgroup.com/services/genotyping), using the competitive allele-specific PCR-KASP® assay.

**Table 1.** SNP used to construct the GRS and the reported traits by genome-wide association studies

Gene and SNP	Effect allele	Lipid trait and effect size in mg/dl (*P* value)								Population and sample size	GWA Study
		HDL-cholesterol		LDL-cholesterol		TAG		TC			
*CETP* rs3764261	A	+0·24	1 × 10^–769^	–0·05	2 × 10^–34^	–0·04	2 × 10^–25^	+0·05	4 × 10^–31^	European ancestry (UK, Finland, Sweden, USA, Italy, Greece, Germany, Estonia, Norway) *n* 94 595	Willer *et al.* (2013)^(72)^
	A	+3·39	7 × 10^–380^			–2·88	1 × 10^–12^	+1·67	7 × 10^–14^	European ancestry (Finland, Sweden, USA, Australia, Iceland, Italy, Netherlands, Germany, UK, Croatia, Switzerland, Austria, France, Denmark) *n* 99 900 for HDL *n* 96 598 for TAG *n* 100 184 for TC	Teslovich *et al.* (2010)^(26)^
	A	+3·48	7 × 10^–29^							Northern Finnish Founder *n* 4763	Sabatti *et al.* (2009)^(65)^
	A	+0·20^*^	9 × 10^–18^							African American *n* 7813	Lettre *et al.* 2011^(63)^
	A	+3·18^*^	7 × 10^–43^							Indian *n* 1036	Khushdeep *et al.* 2019^(66)^
*CETP* rs3764261	A	+6·20	3 × 10^–12^							Japanese *n* 900	Hiura *et al.* 2009^(64)^
*LIPG* rs7241918	G	–1·31	3 × 10^–49^							European ancestry (Finland, Sweden, USA, Australia, Iceland, Italy, Netherlands, Germany, UK, Croatia, Switzerland, Austria, France, Denmark) *n* 99 900	Teslovich *et al.* (2010)^(26)^
	A							–1·94	2 × 10^–19^	European ancestry (Finland, Sweden, USA, Australia, Iceland, Italy, Netherlands, Germany, UK, Croatia, Switzerland, Austria, France, Denmark) *n* 100 184	Teslovich *et al.* (2010)^(26)^
	G	–0·09^*^	1 × 10^–44^					–0·06^*^	4 × 10^–18^	European ancestry (UK, Finland, Sweden, USA, Italy, Greece, Germany, Estonia, Norway) *n* 94 595	Willer *et al.* (2013)^(72)^
*LIPG* rs7241918	G	–0·08^*^	4 × 10^–55^	–0·02^*^	1 × 10^–8^					European ancestry *n* 115 082	Richardson *et al.* (2022)^(84)^
	A	+0·02^*^	3 × 10^–27^							Multi-ancestry (African: *n* 23 761; Asian: *n* 13 171; European: *n* 90 272; Hispanic or Latin American: *n* 6620)	Bentley *et al.* 2019^(71)^
GCKR rs1260326	T					+8·76	6 × 10^–133^	+1·91	7 × 10^–27^	European ancestry (Finland, Sweden, USA, Australia, Iceland, Italy, Netherlands, Germany, UK, Croatia, Switzerland, Austria, France, Denmark) *n* 96 598 for TAG *n* 100 184 for TC	Teslovich *et al.* (2010)^(26)^
	T					+0·12	2 × 10^–239^	+0·05^*^	3 × 10^–42^	European ancestry (UK, Finland, Sweden, USA, Italy, Greece, Germany, Estonia, Norway) *n* 94 595	Willer *et al.* (2013)^(72)^
GCKR rs1260326	T					+0·12^*^	2 × 10^–31^			European (UK, Finland, Sweden, USA, Italy, France) *n* 19 840	Kathiresan *et al.* (2009)^(28)^
	T					+0·12^*^	5 × 10^–88^	+0·05^*^	3 × 10^–13^	European (UK, Finland, Sweden, Iceland, Netherlands, Germany, Estonia) *n* 62 166	Surakka *et al.* (2015)^(85)^
	T			+0·03^*^	6 × 10^–60^					European ancestry *n* 440 546	Richardson *et al.* (2020)^(73)^
	T					1·41^†^	2 × 10^–13^			Mexican *n* 2240	Weissglas-Volkov *et al.* (2013)^(41)^
	T			+0·03^*^	7 × 10^–10^					Multi-ancestry (European: *n* 76 627; Hispanic: *n* 7795; East Asian: *n* 6855; African American: *n* 2958; South Asian: *n* 439)	Hoffman *et al.* 2018^(86)^
*SORT1* rs629301	G			–5·65	1 × 10^–170^			–5·41	6 × 10^–131^	European ancestry (UK, Finland, Sweden, USA, Australia, Iceland, Italy, Netherlands, Germany, Croatia, Switzerland, Austria, France, Denmark) *n* 100 184 for TC *N* 95 454 for LDL-C	Teslovich *et al.* (2010)^(26)^
	G			–0·17^*^	5 × 10^–241^			–0·13^*^	2 × 10^–170^	European ancestry (UK, Finland, Sweden, USA, Italy, Greece, Germany, Estonia, Norway) *n* 94 595	Willer *et al.* (2013)^(72)^
	G	+0·04^*^	4 × 10^–15^					–0·14^*^	7 × 10^–135^	Multi-ancestry (European: *n* 76 627; Hispanic: *n* 7795; East Asian: *n* 6855; African American: *n* 2958; South Asian: *n* 439)	Hoffman *et al.* 2018^(86)^
	T			+4·46^*^	1 × 10^–128^					Multi-ancestry (African: *n* 23 761; Asian: *n* 13 171; European: *n* 90 272; Hispanic or Latin American: *n* 6620)	Bentley *et al.* 2019^(71)^
*SORT1* rs629301	G			–6·03^*^	2 × 10^–72^			–5·80^*^	2 × 10^–57^	European *n* 29 902	Kulminski *et al.* (2020)^(87)^
	T			+0·11^*^	2 × 10^–31^					Japanese *n* 72 866	Sakaue *et al.* (2021) ^(83)^
*LIPC* rs1532085	A	+0·11^*^	1 × 10^–188^					+0·05^*^	7 × 10^–47^	European ancestry (UK, Finland, Sweden, USA, Italy, Greece, Germany, Estonia, Norway) *n* 94 595	Willer *et al.* (2013)^(72)^
	A	+0·11^*^	1 × 10^–213^							Multi-ancestry European: *n* 187 167; East Asian (China, Japan, Republic of Korea, Philippines, Singapore, Taiwan): *n* 34 930	Spracklen *et al.* (2017)^(88)^
*LIPC* rs1532085	G	–0·13^*^	1 × 10^–35^							European ancestry (UK, Finland, Sweden, Australia, Italy, Netherlands, Germany, Croatia, Norway, Denmark) *n* 21 412	Aulchenko *et al.* 2009 ^(74)^
	G					+2·99	2 × 10^–13^			European ancestry (Finland, Sweden, USA, Australia, Iceland, Italy, Netherlands, Germany, UK, Croatia, Switzerland, Austria, France, Denmark) *n* 96 598	Teslovich *et al.* (2010)^(26)^
	A	+1·90	2 × 10^–10^							Northern Finnish Founder *n* 4763	Sabatti *et al.* 2009^(65)^
*APOA1* rs964184	G			+2·85	1 × 10^–26^	+16·95	7 × 10^–240^			European ancestry (UK, Finland, Sweden, USA, Australia, Iceland, Italy, Netherlands, Germany, Croatia, Switzerland, Austria, France, Denmark) *n* 96 598 for TAG; *n* 95 454 for LDL-cholesterol	Teslovich *et al.* (2010)^(26)^
*APOA1* rs964184	G					+0·24^*^	2 × 10^–157^			European (UK, Finland, Sweden, Iceland, Netherlands, Germany, Estonia) *n* 62 166	Surakka *et al.* (2015)^(85)^
	G	–0·03^*^	2 × 10^–11^							European (UK, Finland, Italy, Switzerland) *n* 17 723	Waterworth *et al.* 2010^(76)^
	G	–0·05^*^	3 × 10^–12^			+0·16^*^	4 × 10^–33^			African American: *n* 7601, Hispanic: *n* 3335 for TAG; African American: *n* 7917, Hispanic: *n* 3506 for HDL-cholesterol	Coram *et al.* 2013^(78)^
	G	–0·17	1 × 10^–12^			+0·30^*^	4 × 10^–62^			European ancestry (UK, Finland, Sweden, USA, Italy, France) *n* 19 840	Kathiresan *et al.* (2009)^(28)^

		**CAD**				**MI**					
*ATP2B1* rs2681472	G	1·07^†^	8 × 10^–11^							European *n* 63 731	Nelson *et al.* 2017^(81)^
*ATP2B1* rs2681472	G					+0·07^*^	1 × 10^–11^			European (UK, Finland): *n* 461 823; Japanese: *n* 161 206	Sakaue *et al.* (2021)^(83)^
	G					1·08^†^	6 × 10^–9^			European: *n* 126630, Hispanic or Latin American (USA): *n* 3615, Middle Eastern, North African or Persian: *n* 754, African American or Afro-Caribbean (USA): *n* 2908, South Asian (India, UK, Pakistan): *n* 23 156; East Asian (Republic of Korea, China): *n* 9396	Nikpay *et al.* (2015)^(80)^
	G					1·07^†^	1 × 10^–12^			European *n* ∼472 000	Hartiala *et al.* (2021)^(89)^

SNP, single nucleotide polymorphism; GRS, genetic risk score; TC, total cholesterol; GWA, genome-wide association.

* Effect sizes are in units of SD.

† OR.

### Construction of genetic risk score

To construct the GRS, each SNP was first tested for independent association with the lipid-related traits using linear regression analysis, adjusted for age, sex and BMI. An unweighted GRS was then constructed by summing the number of risk alleles across all the seven SNP (*CETP* rs3764261, *GCKR* rs1260326, *LIPG* rs7241918, *SORT1* rs629301, *LIPC* rs1532085, *APOA1* rs964184 and *ATP2B1* rs2681472) for each participant. For each SNP, a score of 0, 1 or 2 was assigned depending on whether the participant carried no risk alleles (homozygous for the non-risk allele), one risk allele (heterozygote) or two risk alleles (homozygous for the risk allele). The scores for the seven SNP were then added up to create the GRS. The effect sizes of the SNP were not considered and the GRS for each participant represented the total number of risk alleles they carried from the seven SNP. An unweighted GRS was used because although we selected SNP which have shown associations with lipid-related traits, the studies were not conducted in the Brazilian population, and it has been reported that effect sizes may vary across populations and data from a genome-wide association study conducted in one population may not apply to another population^(31,107)^. Moreover, assigning weights to risk alleles has been shown to have minimal effect^(108)^. The risk alleles were defined as alleles previously reported to be associated with increased concentration of TAG, LDL-cholesterol or TC; or reduced concentration of HDL-cholesterol; or increased risk of coronary artery disease or myocardial infarction. The GRS ranged from 1 to 10, and the median GRS (6 risk alleles) was used as a cut-off point for grouping participants as low risk (GRS < 6 risk alleles) or high risk (GRS ≥ 6 risk alleles).

### Statistical analysis

An independent sample t test was used to compare the means of continuous variables between men and women. The results for descriptive statistics are presented as means and sd. To test for normality, the Shapiro–Wilk test was used and all the biochemical, anthropometric and dietary variables, except total fat, carbohydrate, and MUFA intake (percentages of total energy intake (TEI)), were log-transformed prior to the analysis. Allele frequencies were determined by gene counting and Hardy–Weinberg equilibrium was calculated using the Chi-square test. All the seven SNP were in Hardy–Weinberg equilibrium (*P* > 0·05) (online Supplementary Table S1), and the alleles had a frequency >5 %.

Linear regression was used to test the association of the GRS with lipid levels and blood pressure, with adjustment for age, sex and BMI. To determine interactions between the GRS and dietary factors on the outcome variables (TAG, TAG:HDL-cholesterol ratio, HDL-cholesterol, LDL-cholesterol, TC, systolic blood pressure (SBP), and diastolic blood pressure (DBP)), the interaction term was included in the regression model. The dietary factors examined were the intakes of fat, carbohydrate, and protein. Statistically significant GRS–diet interactions (*P* < 0·05) were investigated further by stratifying participants according to the quantity of dietary intake. A significant interaction between the GRS and total fat intake was explored further by analysing the effects of subtypes of fat (SFA, MUFA and PUFA). The median intake of total fat, SFA, MUFA and PUFA was used as a cut-off point to place participants into groups: ‘low’ (for participants with an intake lower than or equal to the median) and ‘high’ (for those with an intake higher than the median); and the effect of the GRS on the outcome was examined for participants in each group. The Bonferroni adjusted *P*-value for association was 0·007 (1GRS * 7 outcome variables = 7 tests; 0·05/7 = 0·007), and for interaction, it was 0·002 (1GRS * 7 outcome variables*3 dietary factors = 21 tests; 0·05/21 = 0·002). The statistical analyses were performed using the Statistical Package for the Social Sciences (SPSS) software (version 28; SPSS Inc., Chicago, IL, USA). Additionally, the GRS was scaled by converting the scores to units of standard deviation from the mean^(109)^ and the association of the GRS as a continuous variable with the lipid-related traits was tested by linear regression using the R software version 4·3·1^(110)^.

#### Power and sample size calculation

Power calculation was performed using the QUANTO software, version 1·2·4 (May 2009)^(111)^ in the form of minimum detectable effect at 80 % power and a significance level of 5 %. For an SNP with a minor allele frequency of 5 %, the minimum detectable effect at 80 % power was 6·6 mg/dl for TC, LDL-cholesterol and TAG. For an SNP with a minor allele frequency of 50 %, the minimum detectable effect at 80 % power was 2·9 mg/dl for TC, LDL-cholesterol and TAG.

## Results

### Characteristics of the study participants

The demographic and clinical characteristics of the participants in this study are summarised in Table 2. The mean age of the sample was 21 ± 2 years, and men had higher BMI and waist circumference than women (*P* = 0·01 and *P* < 0·001, respectively). Women, however, had higher concentrations of HDL-cholesterol (*P* < 0·0001) and TC (*P* = 0·01) but lower TAG:HDL-cholesterol ratio (*P* = 0·006), SBP (*P* < 0·0001), and DBP (*P* < 0·001) than men. Intakes of total energy and protein were higher in men than in women (*P* = 0·003 and *P* = 0·04, respectively), but consumption of total fat, SFA, MUFA, PUFA and carbohydrate did not differ significantly between men and women. Table 3 shows the characteristics of the study participants according to GRS. Participants with a high GRS had a significantly lower intake of energy (*P* = 0·02) than those with a low GRS. No other significant differences were observed between participants in the two groups. The distribution of the GRS across deciles of TC, LDL-cholesterol, TAG and TAG:HDL ratio is presented in online Supplementary Fig. S2.

**Table 2.** Characteristics of study participants by sex

	All (*n* 190)		Women	(*n* 141)	Men	(*n* 49)	*P* Value
	Mean	sd	Mean	sd	Mean	sd	
Age (years)	21	2	21	2	22	2	0·17
BMI (kg/m^2^)	23	1	23	1	24	1	**0·01**
WC (cm)	72	1	69	1	83	1	**<0·001**
TAG (mg/dl)	76	2	76	2	75	2	0·81
TAG:HDL ratio	2	2	1	2	2	2	**0·01**
HDL-cholesterol (mg/dl)	55	1	59	1	46	1	**<0·0001**
LDL-cholesterol (mg/dl)	99	1	100	1	99	1	0·80
TC (mg/dl)	174	1	178	1	163	1	**0·01**
SBP (mmHg)	107	1	105	1	114	1	**<0·0001**
DBP (mmHg)	64	1	63	1	67	1	**<0·001**
Energy (kcal/day)	1735	1	1668	1	1944	1	**0·003**
Total fat (% of energy)	32	6	32	6	31	6	0·14
SFA (% of energy)	9	1	9	1	9	1	0·84
MUFA (% of energy)	8	3	8	3	8	3	0·07
PUFA (% of energy)	5	2	5	2	5	2	0·08
Carbohydrate (% of energy)	51	7	51	7	51	8	0·88
Protein (% of energy)	17	1	16	1	18	1	**0·04**

WC, waist circumference; TC, total cholesterol; SBP, systolic blood pressure; DBP, diastolic blood pressure.

*P* values for the differences in means between men and women were calculated using independent sample *t* test.

**Table 3.** Association of GRS with blood lipids and blood pressure and the characteristics of the participants stratified by GRS

Trait	GRS < 6 (*n* 92)		GRS ≥ 6 (*n* 98)		
	Mean	se	Mean	se	*P* value
TAG (mg/dl)	67·3	1·0	84·9	1·0	**<0·001**
TAG:HDL-cholesterol ratio	1·2	1·0	1·5	1·0	**0·002**
HDL-cholesterol (mg/dl)	54·5	1·0	55·5	1·0	0·56
LDL-cholesterol (mg/dl)	91·4	1·0	107·6	1·0	**<0·0001**
TC (mg/dl)	164·1	1·0	183·7	1·0	**<0·0001**
SBP (mmHg)	106·9	1·0	107·2	1·0	0·69
DBP (mmHg)	63·2	1·0	64·1	1·0	0·48

Characteristic	GRS < 6 (*n* 92)		GRS ≥ 6 (*n* 98)		*P* value^*^
	Mean	sd	Mean	sd	
Age (years)	21	2	21	2	0·28
Sex (W/M)	67/27	–	78/26	–	0·56
BMI (kg/m^2^)	23	1	23	1	0·97
WC (cm)	73	1	72	1	0·59
Energy (kcal/day)	1827	1	1648	1	**0·02**
Total fat (% of energy)	32	6	32	6	0·99
SFA (% of energy)	9	1	9	1	0·45
MUFA (% of energy)	8	2	8	3	0·27
PUFA (% of energy)	5	1	4	2	0·12
Carbohydrate (% of energy)	51	7	50	7	0·68
Protein (% of energy)	17	1	17	1	0·84

GRS, genetic risk score; TC, total cholesterol; SBP, systolic blood pressure; DBP, diastolic blood pressure; W, women; M, men.

*P* values were obtained from linear regression analysis with adjustment for age, sex and BMI. Log-transformed variables were used for the analysis and values in bold represent significant associations.

* *P* values for the differences in means between participants with low GRS and those with high GRS were obtained using independent sample *t* test. The distribution of sex in the two groups was compared using the *χ* ^2^ test.

### Association of the genetic risk score with blood lipids

**Fig. 1.**
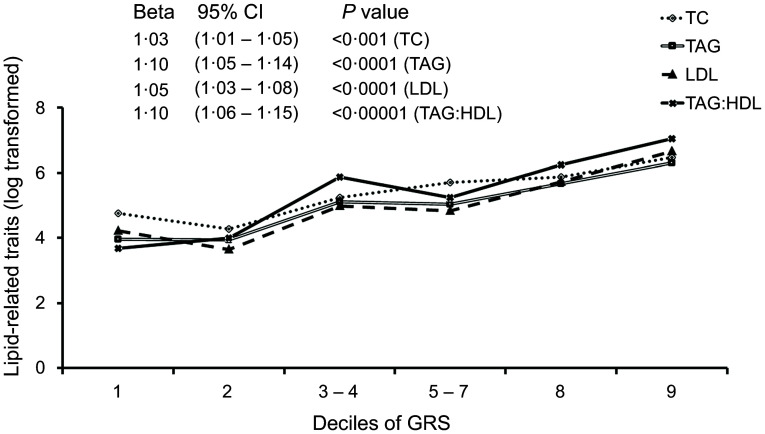
Distribution of lipid-related traits across deciles of GRS (genetic risk score). TC (total cholesterol), LDL-cholesterol (low-density lipoprotein cholesterol), TAG (triacylglycerol), TAG:HDL-cholesterol (TAG to high-density lipoprotein cholesterol ratio). GRS, genetic risk score; TC, total cholesterol.

Four significant associations were identified between the GRS and lipid traits where individuals carrying six or more risk alleles had significantly higher TAG, LDL-cholesterol and TC concentrations, as well as higher TAG:HDL-cholesterol ratio compared with participants with less than six risk alleles (Table 3). When the GRS was tested as a continuous variable, each standard deviation increase in the GRS was associated with a 1·05 mg/dl increase (95 % CI 1·02, 1·07) in the concentration of TC (*P* = 0·002); 1·07 mg/dl increase (95 % CI 1·03, 1·12) in the concentration of LDL-cholesterol (*P* < 0·001); 1·14 mg/dl increase (95 % CI 1·07, 1·21) in the concentration of TAG (*P* < 0·0001) and a 1·16 mg/dl increase (95 % CI 1·09, 1·24) in TAG:HDL-cholesterol ratio (*P* < 0·0001). All the associations remained significant after Bonferroni correction for multiple testing. The distribution of the lipid-related traits across deciles of the GRS is presented in Fig. 1. As the decile of the GRS increased, the concentration of TC, TAG, LDL-cholesterol and TAG:HDL also increased.

**Fig. 2.**
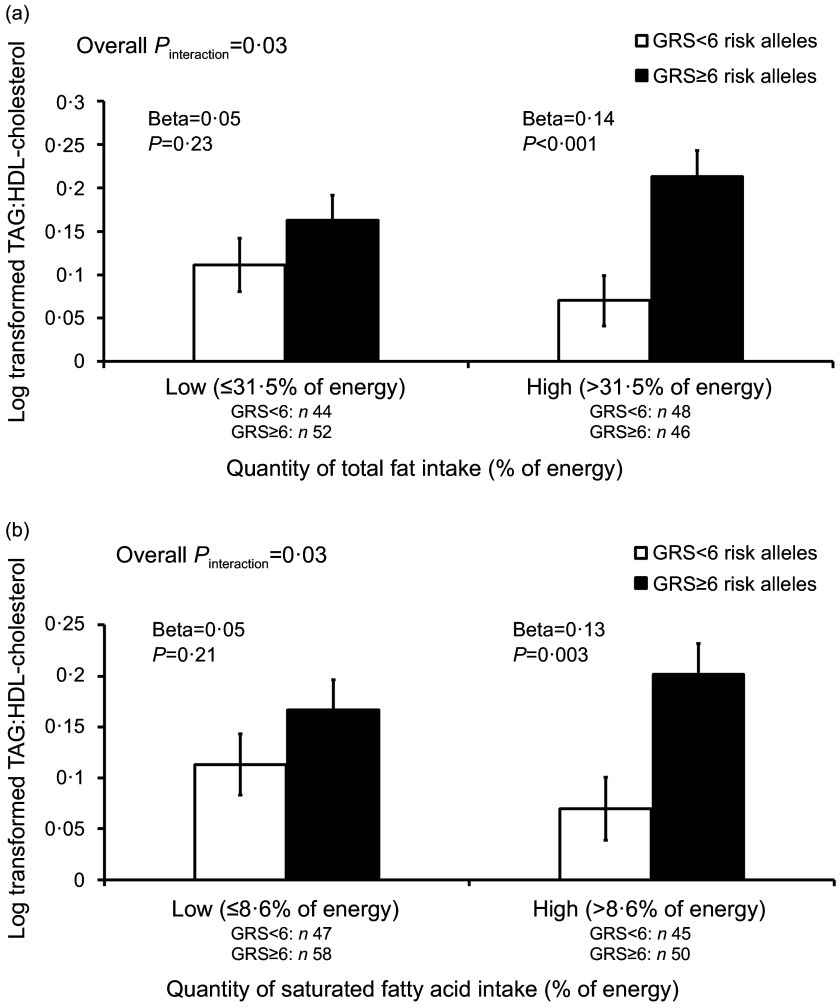
**(a)** Interaction between GRS (genetic risk score) and total fat intake on TAG:HDL-cholesterol (TAG to high-density lipoprotein cholesterol) ratio. Low refers to total fat intake lower or equal to the median and high refers to total fat intake above the median. In the high total fat intake group, participants with a high GRS (≥6 risk alleles) had higher TAG:HDL-cholesterol ratio than those with a low GRS (<6 risk alleles). There was no significant difference in TAG:HDL-cholesterol ratio in the low total fat intake group. **(b)** Interaction between GRS (genetic risk score) and SFA intake on TAG:HDL-cholesterol (TAG to HDL-cholesterol ratio). Low refers to SFA intake lower or equal to the median and high refers to SFA intake above the median. A high intake of SFA was associated with higher TAG:HDL-cholesterol in participants with a high GRS compared with those with a low GRS, but no significant difference in TAG:HDL-cholesterol was observed when SFA intake was low. GRS, genetic risk score.

### Interaction between genetic risk score and dietary factors on blood lipids

There was a significant interaction between GRS and total fat intake on TAG:HDL-cholesterol ratio (*P*_interaction_ = 0·03) as shown in Table 4. In the high total fat intake group (>31·5 % of TEI), participants carrying six or more risk alleles had a higher TAG:HDL-cholesterol ratio compared with those carrying less than six risk alleles (beta = 0·14, 95 % CI: 0·06, 0·23; *P* < 0·001) (Fig. 2(a)). No significant difference in TAG:HDL-cholesterol ratio was found between participants with a high GRS (≥6 risk alleles) and those with a low GRS (<6 risk alleles) in the low total fat intake group (≤31·5 % of TEI). When subtypes of fat were investigated, a significant interaction was found between GRS and SFA intake on TAG:HDL-cholesterol ratio (*P*_interaction_ = 0·03) (Fig. 2(b)), where a high SFA intake (>8·6 % of TEI) was associated with a higher TAG:HDL-cholesterol ratio in participants with a high GRS compared with those with a low GRS (beta = 0·13, 95 % CI: 0·05, 0·22; *P* = 0·003), but there was no significant difference in TAG:HDL-cholesterol ratio when SFA intake was low (≤8·6 % of TEI). A significant interaction was also observed between GRS and total fat intake on HDL-cholesterol concentration (*P*_interaction_ = 0·007). However, when individuals were stratified according to quantity of total fat intake, there was no significant association between the GRS and HDL-cholesterol concentration. The interactions did not pass the Bonferroni threshold.

**Table 4.** Interaction between GRS and dietary factors on blood lipids and blood pressure

Trait	GRS * Protein (% of energy)			GRS * Fat (% of energy)			GRS * Carbohydrate (% of energy)		
	Beta coefficient	se	*P*_interaction_	Beta coefficient	se	*P*_interaction_	Beta coefficient	se	*P*_interaction_
TAG (mg/dl)	0·33	0·30	0·27	0·01	0·01	0·26	–0·004	0·004	0·30
TAG:HDL-cholesterol ratio	0·28	0·32	0·39	0·01	0·01	**0·03**	–0·01	0·004	0·06
HDL-cholesterol (mg/dl)	0·06	0·14	0·70	–0·01	0·002	**0·007**	0·004	0·002	0·05
LDL-cholesterol (mg/dl)	0·29	0·18	0·12	–0·001	0·003	0·75	0·001	0·002	0·69
TC (mg/dl)	0·22	0·13	0·10	–0·002	0·002	0·35	0·001	0·002	0·46
SBP (mmHg)	0·002	0·05	0·96	–0·0002	0·001	0·83	–0·001	0·001	0·17
DBP (mmHg)	–0·03	0·08	0·71	0·00004	0·001	0·98	–0·001	0·001	0·31

GRS, genetic risk score; TC, total cholesterol; SBP, systolic blood pressure; DBP, diastolic blood pressure.

*P* values were obtained from linear regression analysis with adjustment for age, sex and BMI. Log-transformed variables were used for the analysis and values in bold represent significant interactions.

## Discussion

Our findings provide evidence that the genetic risk for disturbances in blood lipids concentration might be modulated by dietary fat intake. Significant interactions were found between the GRS and total fat intake on TAG:HDL-cholesterol ratio and between the GRS and SFA intake on TAG:HDL-cholesterol ratio. Increased consumption of total fat (>31·5 % of energy) and SFA (>8·6 % of energy) was associated with higher TAG:HDL-cholesterol ratio in participants carrying ≥6 risk alleles compared with those with <6 risk alleles. The results suggest that the TAG:HDL ratio in Brazilian young adults with a high genetic risk for disturbances in lipid-related traits maybe responsive to dietary fat intake; hence, interventions targeting a reduction in total fat and SFA intake could potentially benefit these individuals. Although the interactions did not pass the Bonferroni threshold, three of the SNP included in our GRS (*CETP* rs3764261, *APOA1* rs964184 and *GCKR* rs1260326) have previously been reported to interact with dietary fat intake and influence lipid-related traits. In a study involving two trials (a 2-year randomised weight loss trial (POUNDS LOST) consisting of 732 overweight/obese adults and a replication in 171 overweight/obese adults from an independent 2-year randomised weight loss trial (DIRECT))^(112)^, significant interactions were observed between the *CETP* SNP rs3764261 and dietary fat intake on changes in the concentration of HDL-cholesterol and TAG (pooled *P*_interaction_ < 0·01). Similarly, a prospective, randomised, single-blind controlled dietary intervention trial (Coronary Diet Intervention With Olive Oil and Cardiovascular Prevention) involving 424 Spanish individuals with metabolic syndrome^(113)^ found significant interactions between the *CETP* SNP rs3764261 and Mediterranean diet on the concentration of HDL-cholesterol (*P*_interaction_ = 0·006) and TAG (*P*_interaction_ = 0·04). In another study consisting of 734 overweight/obese adults from the POUNDS LOST trial^(114)^, the *APOA1* SNP rs964184 was also found to interact with dietary fat intake in relation to changes in the concentration of HDL-cholesterol, LDL-cholesterol and total cholesterol (*P*_interaction_ = 0·006, 0·02 and 0·007, respectively). Additionally, a cross-sectional study of 3342 individuals (1671 sib pairs) in India^(115)^ found a significant interaction between the *APOA1* SNP rs964184 and dietary fat intake on the concentration of TAG (*P* = 0·04). This study^(115)^ also observed significant interactions between the *CETP* SNP rs3764261 and dietary fat intake on the concentrations of total cholesterol (*P* = 0·02) and LDL-cholesterol (*P* = 0·04). Furthermore, an interaction between the *GCKR* SNP rs1260326 and MUFA intake on HDL-cholesterol concentration was reported in a cross-sectional study of 101 participants of different ethnicities in the USA population (*P*_interaction_ = 0·02)^(116)^. Therefore, the interactions in our study cannot be ruled out completely; hence, a replication is warranted.

The ratio of TAG:HDL-cholesterol has been identified as an independent predictor of CHD, mortality from CVD and insulin resistance^(16,17,117,118)^. Hence, our findings have significant public health implications in terms of prevention and management of dyslipidaemia in individuals with a high genetic risk. Our data support the recommendations of the WHO^(14)^ to reduce the intake of total fat and SFA to less than 30 % and 10 % of energy intake, respectively, to help prevent cardiometabolic diseases. Our findings are also in agreement with the dietary guidelines for Brazilians which recommend decreasing the intake of food rich in solid fat and added sugar and limiting the daily energy intake from total fat to less than 30 %^(119,120)^.

In the current study, the GRS was positively associated with the concentration of TAG, LDL-cholesterol and TC and the ratio of TAG:HDL-cholesterol. Our findings are consistent with those of a study involving 8526 participants from two Danish cohorts^(121)^ (a randomised nonpharmacological intervention study (Inter99), *n* 5961; and a population-based epidemiological study (Health2006), *n* 2565), in which a positive association was identified between lipid-GRS and the concentration of TAG (beta = 1·4 % mmol/l, *P* < 0·0001); LDL-cholesterol (beta = 0·024 mmol/l, *P* < 0·0001) and TC (beta = 0·027 mmol/l, *P* < 0·0001). Similarly, a prospective study of 3495 Swedish participants^(122)^ reported significant associations between lipid-GRS and changes in the concentration of TC and TAG after a 10-year follow up (beta = 0·02 mmol/l per effect allele, *P* < 0·0001 for TC; beta = 0·02 mmol/l per effect allele, *P* < 0·0001 for TAG). The European Prospective Investigation of Cancer-Norfolk cohort study, consisting of 20 074 participants^(123)^, also found a positive association between a lipid-GRS and the concentration of TAG (beta = 0·25 mmol/l, 95 % CI 0·22, 0·27 per allele change; *P* < 0·001), indicating the role of genetic polymorphisms in predicting variability in blood lipid concentration.

A systematic review and meta-analysis of six prospective studies including 10 222 participants^(16)^ reported that, in patients with CHD, those with elevated TAG:HDL-cholesterol ratio had increased risk of all-cause mortality (hazard ratio = 2·92, 95 % CI 1·75, 4·86; *P* < 0·05) and major adverse cardiovascular events (hazard ratio = 1·56, 95 % CI 1·11, 2·18; *P* < 0·05) compared with those with lower TAG:HDL-cholesterol ratio. In line with our findings, a study conducted in 228 Brazilian adults^(50)^ reported a significant interaction between a GRS based on lipid metabolism genes and intake of solid fat, alcoholic beverages and added sugar on the risk of dyslipidaemia (*P*_interaction_ < 0·001), where participants with a high GRS had a lower risk of dyslipidaemia when their intake of solid fat, alcoholic beverages and added sugar was below the median. Similarly, a prospective randomised controlled trial involving 523 Spanish patients with coronary artery disease from the Coronary Diet Intervention With Olive Oil and Cardiovascular Prevention study^(124)^ reported that, carriers of the risk allele (‘G’ allele) of *APOA1* SNP rs964184 who consumed a low-fat diet (containing <30 % of total fat) had reduced post-prandial TAG concentrations after 3 years, while ‘G’ allele carriers on a Mediterranean diet (containing a minimum of 35 % of total fat) continued to have higher post-prandial TAG concentrations. Along these lines, a fat response genetic score based on SNP showing a positive interaction with dietary fat in relation to LDL-cholesterol was found to predict a 1-year change in LDL-cholesterol in a sample of 422 Black and Hispanic participants from the Women’s Health Initiative cohort^(125)^. A significant interaction was identified between the dietary modification trial arm and fat response genetic score for LDL-cholesterol concentration (*P* = 0·002), where participants in the control arm showed a trend towards minimal reductions in LDL-cholesterol concentrations at higher fat response genetic scores, while the opposite trend was observed in participants following a low-fat diet^(125)^. Taking together, these findings suggest that the genetic susceptibility to dyslipidaemia could be modulated by dietary fat intake in different populations.

A nationwide dietary survey involving 32 749 Brazilian individuals (≥10 years old)^(126)^ highlighted a change in dietary pattern in Brazil which is characterised by increased consumption of processed foods rich in fat and simple sugars. An increase in the consumption of ultra-processed food among Brazilians aged ≥10 years was also reported in a study using food consumption data from 2008–2009 (*n* 34 003) to 2017–2018 (*n* 46 164) Household Budget Surveys^(127)^. Similarly, an assessment of the diet quality of Brazilians using data from the national survey^(119)^ showed that, in 60 % of the population, the mean SFA intake was 10·7 % of TEI, which exceeds the WHO’s recommendation of <10 % of TEI^(14)^. The study^(119)^ also reported that solid fat and added sugar contributed more than 45 % of TEI. In the present study, the median intake of total fat was 31·5 % of TEI which is more than the recommended intake of <30 %^(14)^; however, the median intake of SFA (8·6 % TEI) was within the recommended level^(14)^. This suggests that individuals who have a genetic predisposition to dyslipidaemia may find greater benefit from adhering to dietary recommendations.

The mechanisms through which dietary fat intake affects blood lipid concentration have been examined by several studies^(12,128–131)^. Dietary fatty acids affect lipid metabolism through the activation of several transcription factors and nuclear receptors including PPAR and liver X receptors^(128,131)^. PPAR regulate the expression of different genes involved in lipid and lipoprotein metabolism, and the activation of PPAR is positively correlated with the chain length and degree of unsaturation of fatty acids^(12,128,131)^. SFA are also believed to decrease LDL-cholesterol receptor activity which slows the clearance of TAG-rich lipoproteins^(128)^, and this could explain the increased TAG:HDL-cholesterol ratio observed among participants in the high SFA intake group. Consumption of SFA has also been shown to suppress the expression of genes involved in fatty acid oxidation and synthesis of TAG^(12)^ and promote the expression of inflammatory genes^(132)^. However, SFA of different chain lengths and from different food sources have been reported to exert different effects on cardiometabolic traits^(133,134)^.

The main strength of our study is the use of a GRS based on established lipid metabolism genes. Our study is one of few studies which have utilised this approach to explore CVD traits in Brazilian young adults, considering the increased prevalence of CVD in young people aged 15–49 years in Brazil in 2019^(135)^. The GRS approach is more effective in assessing the genetic contribution to complex traits such as blood lipid concentration since single variants often have moderate effect sizes and hence less likely to accurately predict the genetic risk of multifactorial traits^(11,35,136)^. Another strength is the use of validated techniques and trained personnel to assess biochemical, anthropometric and dietary variables, which enhances the accuracy of the assessments. However, our study has some limitations. The small sample size could have influenced our findings since large sample sizes improve the power to detect interactions with small effects^(137,138)^. Given that we did not have access to another Brazilian young adult cohort, we were not able to replicate our study findings. However, we were able to replicate previously reported associations and interactions. Another limitation is the use of self-reported dietary recalls that can introduce bias through overestimation and underestimation of dietary intake^(139,140)^. Moreover, we did not investigate types or food sources of SFA, which have been reported to have different effects on CVD traits^(133,141)^. Additionally, the cross-sectional design means that causality between dietary fat intake and TAG:HDL-cholesterol ratio cannot be established^(31)^.

In conclusion, our study provides evidence that the genetic risk of increased TAG:HDL-cholesterol ratio might be modulated by dietary fat intake. The findings indicate that Brazilian young adults with a high genetic risk for dyslipidaemia might benefit from limiting their intake of total fat and SFA. Our results support the dietary guidelines of the WHO which recommend reducing total fat and SFA to help prevent cardiometabolic diseases. The findings suggest that personalised nutrition strategies based on GRS might be effective for the prevention and management of dyslipidaemia but confirmation in dietary intervention studies with large sample sizes is required.

## Supporting information

Wuni et al. supplementary material 1 Wuni et al. supplementary material

Wuni et al. supplementary material 2 Wuni et al. supplementary material

Wuni et al. supplementary material 3 Wuni et al. supplementary material

Wuni et al. supplementary material 4 Wuni et al. supplementary material

Wuni et al. supplementary material 5 Wuni et al. supplementary material

Wuni et al. supplementary material 6 Wuni et al. supplementary material

## Supplementary material

For supplementary material/s referred to in this article, please visit https://doi.org/10.1017/S0007114524001594
