# Supplementary figures and images for: Interaction between genetic risk score and dietary fat intake on lipid-related traits in Brazilian young adults
Source: Br J Nutr. 2024 Sep 23;132(5):575–89. doi: 10.1017/S0007114524001594 (PMC11536265; doi:10.1017/S0007114524001594)

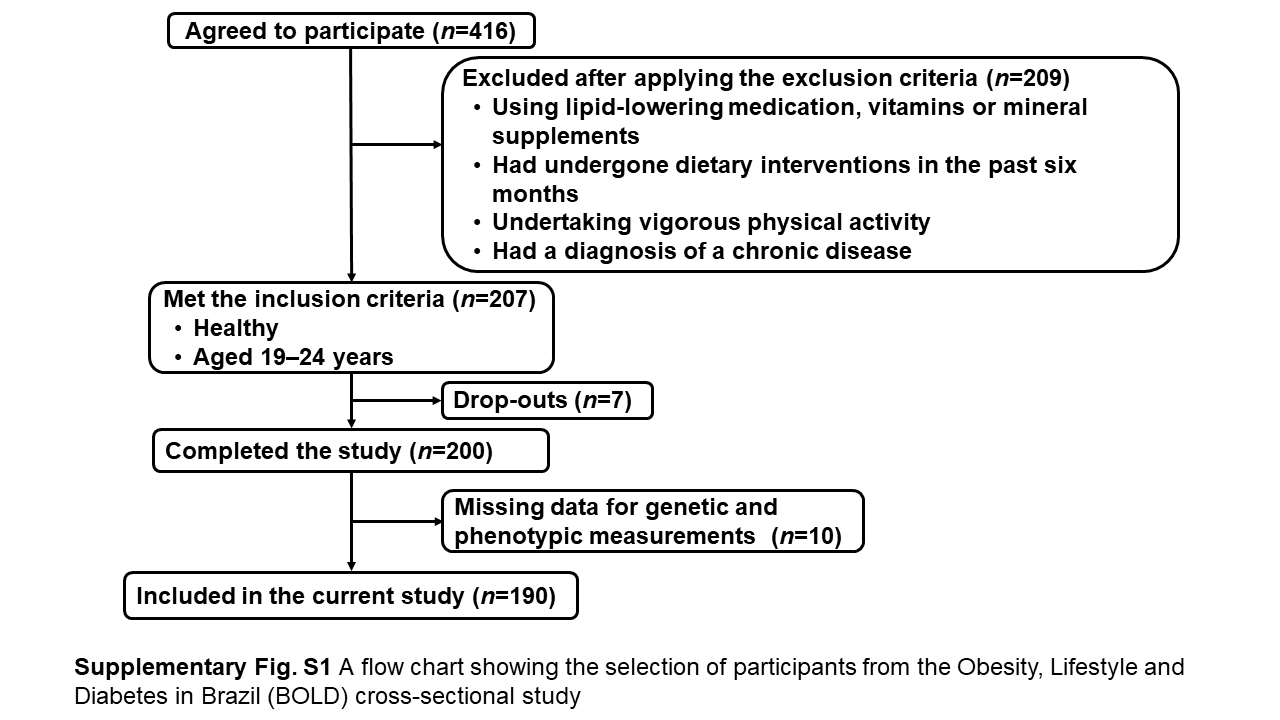

Supplement: Wuni et al. supplementary material 1 — Wuni et al. supplementary material [file S0007114524001594sup001.TIF]

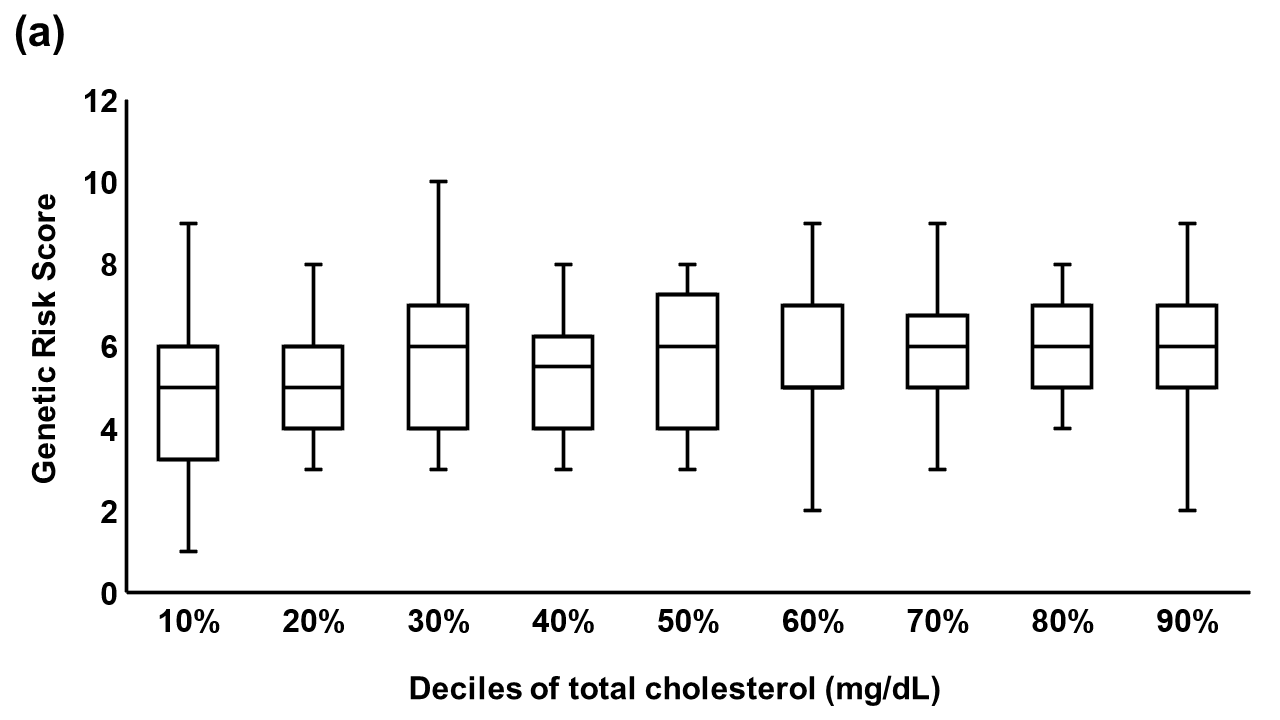

Supplement: Wuni et al. supplementary material 2 — Wuni et al. supplementary material [file S0007114524001594sup002.TIF]

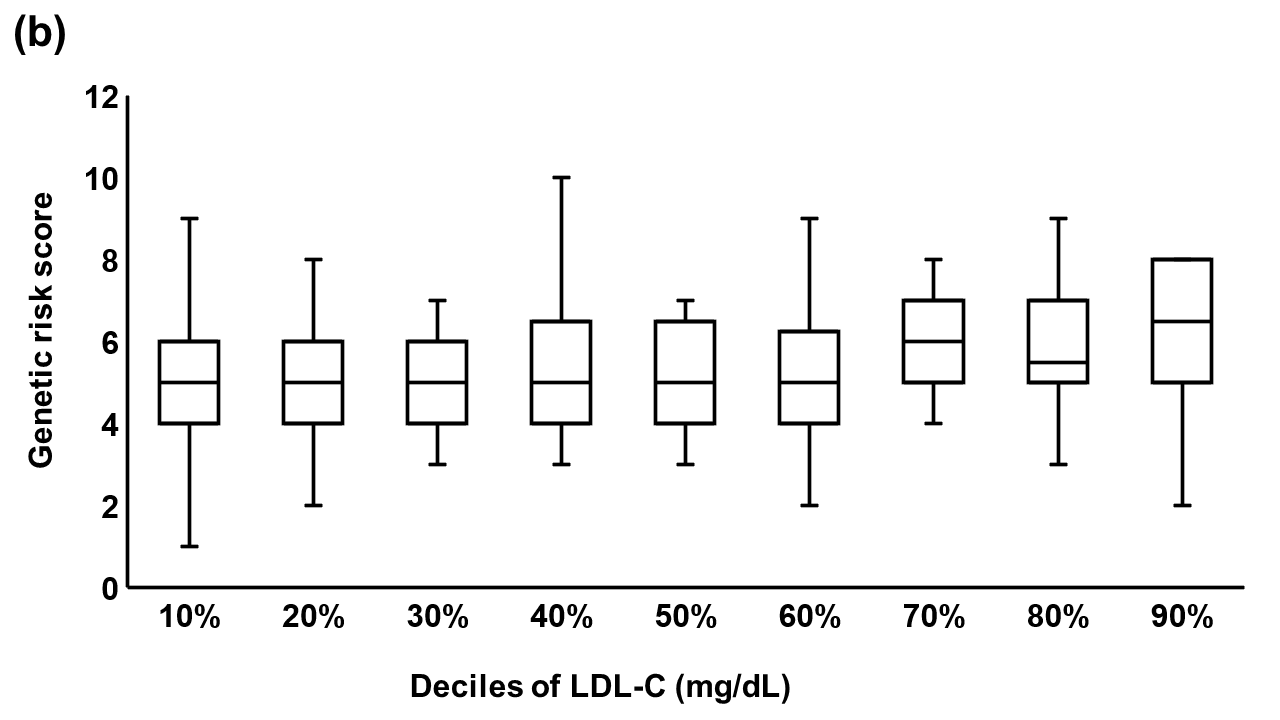

Supplement: Wuni et al. supplementary material 3 — Wuni et al. supplementary material [file S0007114524001594sup003.TIF]

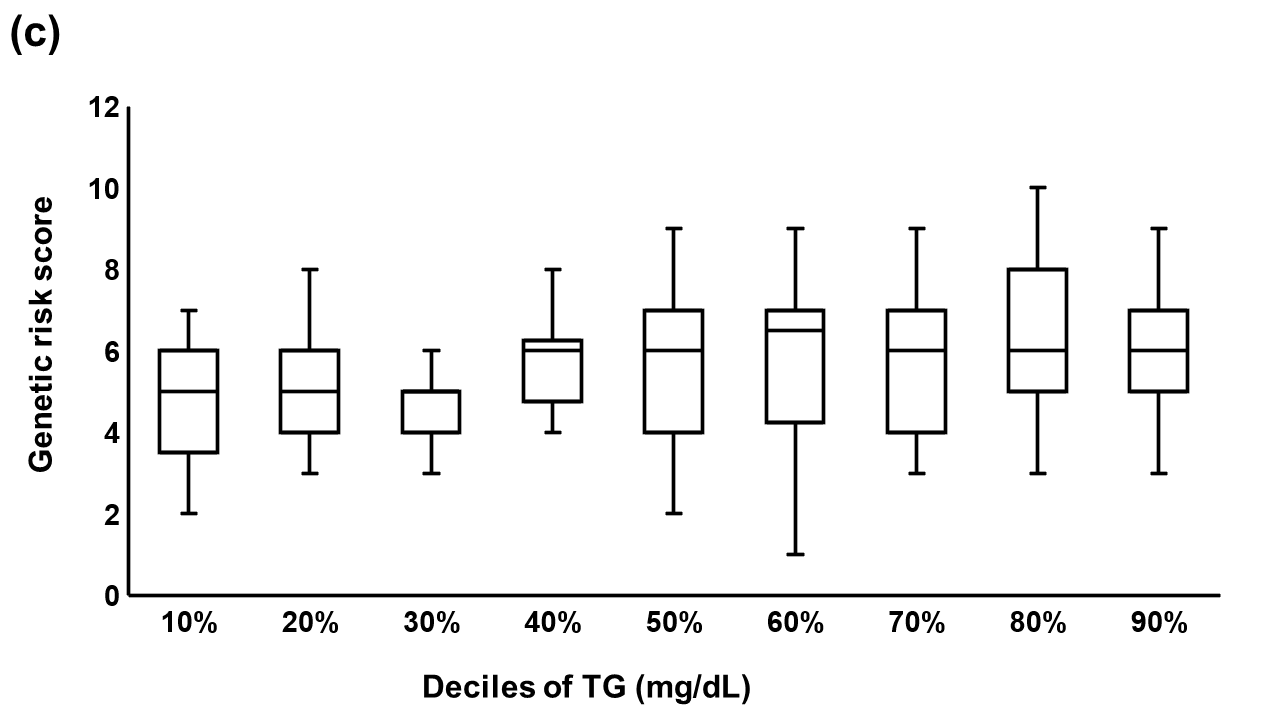

Supplement: Wuni et al. supplementary material 4 — Wuni et al. supplementary material [file S0007114524001594sup004.TIF]

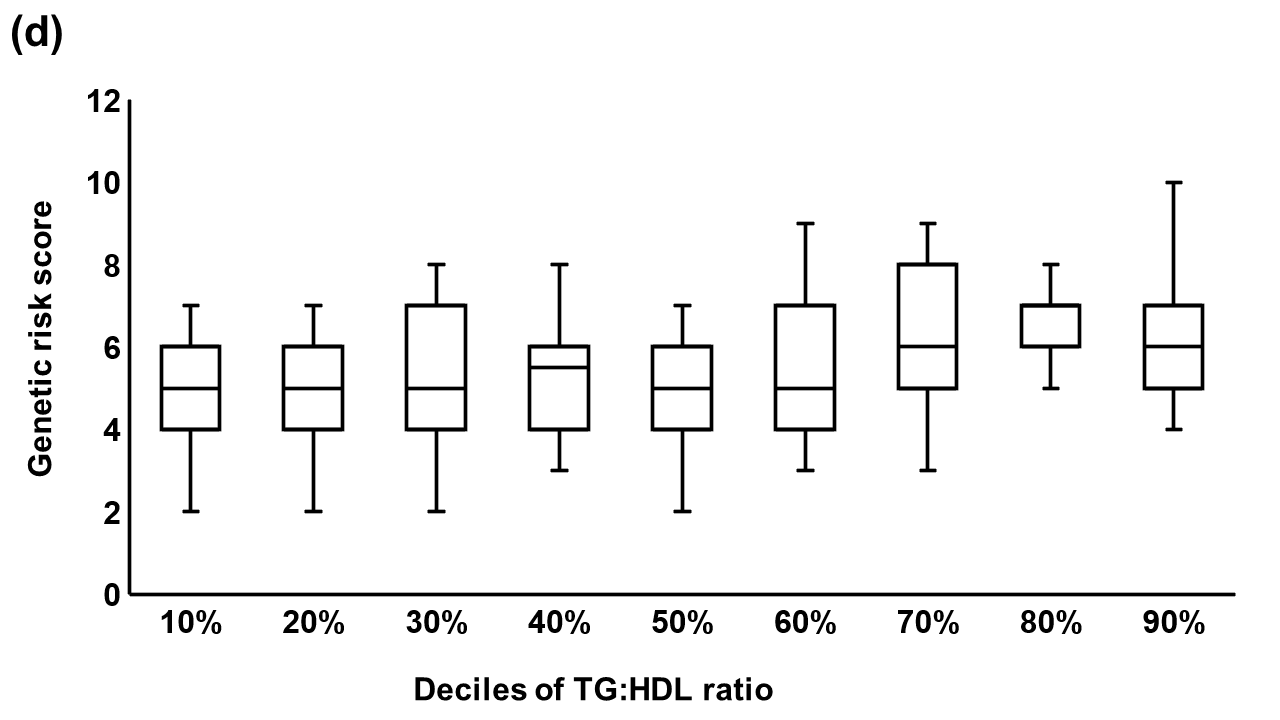

Supplement: Wuni et al. supplementary material 5 — Wuni et al. supplementary material [file S0007114524001594sup005.TIF]
