# Supplementary material for: Interaction between genetic risk score and dietary fat intake on lipid-related traits in Brazilian young adults
Source: Br J Nutr. 2024 Sep 23;132(5):575–89. doi: 10.1017/S0007114524001594 (PMC11536265; doi:10.1017/S0007114524001594)
Supplement: Wuni et al. supplementary material 6 — Wuni et al. supplementary material [file S0007114524001594sup006.docx]

**Table S1** Allele Frequencies and Hardy-Weinberg Equilibrium P-value (n=190)

| **Gene & SNP** | **Genotype Count** | **Allele Frequency in this study (%)** | **Hardy-Weinberg Equilibrium *P*-value** |
| --- | --- | --- | --- |
|  |  |  |  |
| *CETP* rs3764261 | GG = 107  GT = 70  TT = 13 | G = 75  T = 25 | 0.74 |
| *GCKR* rs1260326 | CC = 82  CT = 81  TT = 27 | C = 64  T = 36 | 0.34 |
| *LIPG* rs7241918 | TT = 150  TG = 36  GG = 4 | T = 88  G = 12 | 0.30 |
| *SORT1* rs629301 | TT = 117  TG = 59  GG = 14 | T = 77  G = 23 | 0.10 |
| *LIPC* rs1532085 | GG = 67  GA = 91  AA = 32 | G = 59  A = 41 | 0.91 |
| *APOA1* rs964184 | CC = 115  CG = 68  GG = 7 | C = 78  G = 22 | 0.43 |
| *ATP2B1* rs2681472 | AA = 134  AG = 53  GG = 3 | A = 84  G = 16 | 0.38 |
| SNP, single nucleotide polymorphism; *CETP*, cholesteryl ester transfer protein; *GCKR*, glucokinase regulator; *LIPG*, endothelial lipase; *SORT1*, sortilin 1; *LIPC*, hepatic lipase; *APOA1*, apolipoprotein A1; *ATP2B1*, ATPase plasma membrane Ca2+ transporting 1. | | | |
